# Possible Retinal Impairment Secondary to Ritonavir Use in SARS-CoV-2 Patients: A Narrative Systematic Review

**DOI:** 10.1155/2020/5350494

**Published:** 2020-08-22

**Authors:** Grazia Maria Cozzupoli, Maria Cristina Savastano, Benedetto Falsini, Alfonso Savastano, Stanislao Rizzo

**Affiliations:** ^1^UOC Oculistica, Fondazione Policlinico Universitario A. Gemelli IRCCS, Rome 00168, Italy; ^2^Università Cattolica del Sacro Cuore, Rome 00168, Italy; ^3^Consiglio Nazionale delle Ricerche, Istituto di Neuroscienze, Pisa 56124, Italy

## Abstract

Some reports described a possible ritonavir-related retinal toxicity. The objective of this research was to review and analyze previous studies conducted on ritonavir administration and retinal impairment in a narrative synthesis. PubMed was used to perform a systematic review of ritonavir effects and retinal damage. All studies up to December 2019 were considered. Seven single cases and one case series, reporting a total of 10 patients affected by retinal changes secondary to long-term ritonavir treatment, were included in the review. Variable degrees of outer retina and retinal pigment epithelium changes were detected in most of the patients, with two patients showing macular telangiectasia, four patients presenting intraretinal crystal deposits, two patients disclosing a bull's eye maculopathy, and two patients revealing midperipheral bone spicule-like pigment changes. In the present study, we hypothesized that the use of ritonavir in life-saving treatments of severe acute respiratory syndrome-coronavirus-2 (SARS-CoV-2) pneumonia might expose these patients to the risk of developing a retinotoxicity. We aimed to alert ophthalmologists on the importance of recognizing ritonavir-induced retinal impairment in SARS-CoV-2 patients. These findings are the target for personalized medicine.

## 1. Introduction

Ritonavir is a human immunodeficiency virus type 1 (HIV-1) protease inhibitor, approved by the Food and Drug Administration in 1996 to be used in the highly active antiretroviral therapy (HAART), which is recognized as the most effective treatment method for human immunodeficiency virus (HIV) patients.

In light of the benefits produced by ritonavir in combination with lopinavir in the treatment of severe acute respiratory syndrome and Middle East respiratory syndrome, the association lopinavir/ritonavir has been integrated in the treatment guidelines of 2019 novel coronavirus (2019-nCoV) infected pneumonia [1] with a weak recommendation level [2]. Although a recent randomized trial [3] involving hospitalized adult patients with confirmed severe acute respiratory syndrome-coronavirus-2 (SARS-CoV-2) infection has not shown significant benefits with lopinavir/ritonavir treatment beyond standard care, this protease inhibitor association is used in worldwide hospitals [4].

### 1.1. Mechanism of Action

As an HIV-1 protease inhibitor, ritonavir prevents the cleavage process of viral polyprotein precursors into mature and functional proteins, hence interrupting the production of new viral particles. Although ritonavir was initially designed to inhibit HIV-1 protease, studies have found that it also inhibits cytochrome P450-3A4 (CYP450-3A4) and, to a lesser extent, cytochrome P450-2D6 (CYP450-2D6) [5, 6]. The CYP450-3A4 pathway metabolizes other protease inhibitors. For this reason, coadministration of low-dose ritonavir with other protease inhibitors enhances their bioavailability and allows lower dosing and less frequent administration of these drugs [5].

The most common side effects of ritonavir are nausea, vomiting, diarrhea, change in taste, fatigue, rash, and, with long-term use, hyperlipidemia and lipodystrophy. Retinal toxicity has been described by some authors in case reports and series, but it is a rare and little-known complication of ritonavir. We review the side effects of ritonavir in the human retina.

### 1.2. Ritonavir-Induced Retinal Toxicity

To the best of our knowledge, this is the first review of the retinal changes caused by ritonavir in HAART-treated patients. Although retinotoxicity secondary to ritonavir has not yet been investigated by pharmacokinetic and pharmacodynamic studies on humans, animal data submitted to the European Medicines Agency [7] have identified liver, retina, thyroid, and kidney as susceptible to dose-related ritonavir damage. Moreover, the same report described hypertrophy of the retinal pigment epithelium (RPE) and retinal degeneration in rodents treated with ritonavir and assumed that rodent liver and eye lesions due to ritonavir were related to phospholipidosis. In fact, the electron micrographs of both liver parenchyma and retina demonstrated the presence of amorphous granular inclusion bodies, characteristic of phospholipidosis, and this phenomenon appeared more predominant in the retina than in the liver.

In addition, a study [8] conducted on *in vitro* cellular models demonstrated that ritonavir can strongly inhibit hypoxia-inducible factor 1*α* and vascular endothelial growth factor (VEGF). Interestingly, VEGF knockout mice exhibit degeneration of the choriocapillaris-RPE complex and dysfunction of cone photoreceptors [9]. On the basis of this evidence, Kurihara et al. [9] postulated that VEGF might have a direct neurotrophic effect on photoreceptors in nonhypoxic retinas in addition to the well-known role in regulating endothelial cell function [10]. Therefore, VEGF inhibition by ritonavir should be considered as a further pathogenetic mechanism underlying the toxic damage to the choriocapillaris, RPE cells, and photoreceptors.

## 2. Methods

Because of the shortage of reports found in PubMed about the association between ritonavir and retinal toxicity, we adhered to the Preferred Items for Systematic Reviews and Meta-Analyses (PRISMA) guidelines [11], but we did not need to create a flow diagram.

### 2.1. Study Selection

A literature search was carried out on PubMed and The Cochrane Library. The search query included ritonavir AND (retina OR ocular OR retinal) AND (toxicity OR toxic OR changes OR impairment OR damage). The inclusion criteria for each study were as follows: [1] documented retinal impairment due to ritonavir treatment and [2] conducted in human eyes. All full-text selection was carried out by two authors (G.M.C. and M.C.S.) independently. Any disagreements were resolved by consensus with a third author (B.F).

### 2.2. Data Collection and Analysis

The small number of reference reports did not allow any statistical analyses for data correlation or comparison. However, the qualitative features of the retinal impairment were reported in detail in each included study. Thus, we conducted a narrative systematic review of the published reports describing the clinical features and imaging signs associated with ritonavir-induced retinal toxicity.

In order to establish the macular atrophy stage, we followed the new consensus definition for atrophy associated with age-related macular degeneration on OCT [12]. Images from each report were analyzed and graded by two readers (G.M.C. and M.C.S.) separately. In case of disagreement, a third author (B.F.) was consulted to reach consensus.

The quality of the reports was assessed using the Joanna Briggs Institute Critical Appraisal Checklist for case reports [13]. Two reviewers (G.M.C. and M.C.S.) rated the studies independently, and the final decision was reached by consensus with a third author (B.F.).

## 3. Results and Discussion

### 3.1. Results

The literature search yielded 9 potentially relevant publications. A total of 8 papers satisfied the predetermined inclusion criteria and were included in the review, 7 of which were single case reports and 1 was a case series including 3 patients. One case report was excluded since it did not provide exhaustive clinical details and imaging (Appendix A). The mean score of the Joanna Briggs Institute Critical Appraisal Checklist was 6. Overall, 10 patients affected by retinal abnormalities secondary to long-term ritonavir treatment were imaged and reported between 2011 and 2019 (Table 1). Ritonavir was administered at a dose of 100 mg daily or twice daily [14, 15, 20] in the context of a HAART regimen. The overall average treatment duration was 75 months. The mean age of the patients was 47 years and the patients were all male.

All reports dealing with chronic ritonavir-induced retinal toxicity were united by a common feature, which is the presence of macular pigmentary changes and outer retina impairment. In almost all described cases, ritonavir-related retinotoxicity presented at the fundus examination as a bilateral mottling or atrophy of the RPE affecting the macula, with unilateral macular involvement in one case [18] and involvement of either the macula and the peripheral retina in three cases [17, 20, 21]. Moreover, some peculiar clinical pictures were reported by different authors. Four cases were similar to the clinical picture of type 2 macular telangiectasia (Mac Tel type 2), two of which [14] presenting macular capillary telangiectasias and crystalline deposits, one other [14] revealing extensive macular atrophy, intraretinal cysts, and crystal deposits, and the fourth one [19] showing macular graying and both crystalline and pigment deposits, without evident telangiectasias.

Two authors reported a bull's eye macular pattern [15, 21]. In particular, Louie and Jones [21] described a patient with bull's eye maculopathy associated with bone spicule-like pigment changes in the midperipheral retina. This latter finding was observed as well in a case presenting as a pseudoretinitis pigmentosa [17].

No alterations of the optic disk and retinal vessels were found. The main optical coherence tomography (OCT) features shown by these patients were as follows:

1. Complete RPE and outer retinal atrophy (cRORA) defined by a zone of homogeneous choroidal hypertransmission and absence of the RPE band with overlying outer retinal thinning and loss of photoreceptors [16, 19, 20].
2. Incomplete RPE and outer retinal atrophy (iRORA), with some discontinuous hypertransmission visible but irregular RPE band and photoreceptor degeneration [17, 19].
3. Incomplete and complete outer retinal atrophy (iORA and cORA) characterized by visible and nonvisible external limiting membrane and ellipsoid zone, respectively, and paralleled with nondetectable interdigitation zone and severe thinning of the outer retina in the setting of an intact RPE band [18].
4. Intraretinal cysts and cavitations [14].
5. Epiretinal membrane (ERM) and full-thickness macular hole (FTMH) [20].

Corresponding to the areas of RPE atrophy, the fundus autofluorescence (FAF) showed a patchy hypo-autofluorescence (hypo-AF), surrounded by a speckled or annular hyper-autofluorescence (hyper-AF), attributed to the active toxicity of ritonavir [16, 20].

### 3.2. Discussion

As far as we are aware, this is the first systematic review of the literature concerning ritonavir-induced retinal changes.

There are many antiviral treatments currently used against SARS-CoV-2 infection for which ocular side effects (mainly related to conditions other that direct cellular toxicity, such as inflammatory responses, autoimmunity reactivation, and vascular damage) have been pointed out [22]. Unlike these antivirals, ritonavir appears to exert a toxic effect on the outer retina and RPE, causing their irreversible degeneration in the long term and threatening the central vision without therapeutic options available. Thus, we aimed to alert ophthalmologists on the possible retinal damage that might occur in some patients hospitalized for SARS-CoV-2 pneumonia and treated with ritonavir.

Indeed, we do not know yet if the administration time of life-saving therapies containing ritonavir in SARS-CoV-2-infected patients is sufficiently long to produce a retinal impairment. The overall average treatment duration in the reported cases of HIV patients was 75 months in the context of a HAART regimen. Differently, in the Chinese trial cited above [3], SARS-CoV-2 patients received lopinavir-ritonavir (400 mg and 100 mg, respectively) twice a day for 14 days. Given the brief duration of ritonavir treatment in SARS-CoV-2 patients, it is unlikely that patients will develop a retinal toxicity, which is supposed to be dose- and time-related [23]. Nonetheless, some polymorphisms, for example, on the genes encoding enzymes involved in drug hepatic clearance, and certain comorbidities could place specific subsets of patients at an increased risk for retinal changes. SARS-CoV-2 infection has been shown to involve mainly the respiratory system but also the liver and kidneys [24–26], impairing the metabolism and excretion of the medications used to treat the disease. On the one hand, liver dysfunction can reduce ritonavir hepatic clearance, increasing the total serum drug concentration and predisposing to more severe side effects. On the other hand, kidney injury can impair excretion, dosing, and expected concentrations of hydroxychloroquine, another drug currently used for the treatment of SARS-CoV-2 infection [27, 28] and given together with antiviral and supportive therapies. Hydroxychloroquine has a well-known dose- and time-related toxicity on RPE cells and photoreceptors [29, 30]. Furthermore, a case of short-term retinal toxicity induced by hydroxychloroquine has been recently described by Pasaoglu and Onmez [31]. Anyway, it is not illogical to hypothesize that the retinal toxic effects of both long-term hydroxychloroquine [32] and ritonavir therapies might appear also after short-term treatments, as in the case of SARS-CoV-2 patients, enhanced by the deterioration of renal and hepatic clearance function, respectively. Noteworthily, we cannot exclude that even subtle toxic changes caused by short-term hydroxychloroquine and ritonavir, independently, may add up because of their coadministration, giving a clinically significative picture in the retina, as alerted by Romano et al. [33]. Last, the most vulnerable to SARS-CoV-2 infection and complications are people aged 65 years and older [34]. The greater prevalence of age-related macular degeneration in this age group [35] can represent a further risk factor for the development of toxic maculopathy.

Although short-term toxicity by ritonavir is far less likely than a long-term impairment, we speculate that in the future we might see some retinopathies in which the role of ritonavir toxicity cannot be ruled out. In similar cases, it would be difficult to discriminate between ritonavir toxic sequelae, possible direct SARS-CoV-2 damage due to its particular ocular tropism [36], and consequences of coadministered hydroxychloroquine.

## 4. Conclusion

This systematic review provides a comprehensive summary of the retinal toxic changes secondary to ritonavir treatment. It might be useful to raise the awareness of patients treated by ritonavir for SARS-CoV-2 infection about the possibility of sight-threatening side effects related to the received therapy so that they can refer to a retina specialist for the research of early signs of retinotoxicity in line with the aims of personalized medicine.

## Figures and Tables

**Table 1:** Demographic and clinical characteristics of the reported patients.

Authors	Number of patients	Age (y)	Sex	Treatment duration (m)	Retinal involvement	Fundoscopy	FAF	OCT	Liver dysfunction	ERG	FUP duration (m) and findings
Roe et al. [14]	3	40 to 46	M	19 to 60	Bilateral macular	(i) RPE hypertrophic and atrophic changes (diffuse retinal pigment epitheliopathy) (ii) Parafoveal opacification (iii) Intraretinal crystal deposits	Sharply edged hypo-AF corresponding to the RPE atrophy areas	(i) Intraretinal cysts (ii) Intraretinal cavitations (iii) RPE irregularity (iv) cRORA	Present	—	(i) 12 to 24 m (ii) Decreased visual acuity (iii) Significantly larger areas of RPE disruption (even despite treatment interruption in patient 1) (iv) Evident intraretinal crystals

Pinto et al. [15]	1	30	M	60 (with 24-month interruption)	Bilateral macular	Perimacular ring of pigment mottling, with clumps of pigment in the adjacent periphery	Area of annular hypo-AF (bull's eye maculopathy) with a surrounding ring of hyper-AF	Macular thinning	Absent	ff-ERG: normal	—

Biancardi and Curi [16]	1	—	—	Long term (not specified)	Bilateral macular	Bilateral rounded hypopigmented lesions	(i) Background granularity (LE > RE) (ii) Hypo-AF (RE > LE) (iii) Speckled hyper-AF pattern surrounding the hypo-AF areas	(i) Subfoveal cRORA (ii) Perifoveal areas of hyperreflectivity affecting the ONL and the ELM	—	—	(i) 8 m (ii) Reduction of background granularity and hyper-AF pattern and increase in hypo-AF areas of RPE atrophy

Papavasileiou et al. [17]	1	59	M	96	Bilateral retinal and macular	Bilateral retinitis pigmentosa-like appearance of the fundus with scattered bone specula pigmentation in the midperipheral retina	Paramacular mottled hypo-AF, affecting the macula of the RE and sparing the macula of the LE	iRORA with sparing of the foveolar ellipsoid zone in the LE	Absent	(i) PERG P50: undetectable (ii) ff-ERG: rod-cone dystrophy with additional inner retinal involvement	—
Tu et al. [18]	1	47	M	84	Unilateral macular (LE)	Unilateral hyperemic lesion centered at the left fovea	Normal	Subfoveal iORA and parafoveal cORA	Present	—	(i) 6 m; improved visual acuity and epitheliopathy resolution already after 6 weeks of ritonavir discontinuation (ii) Stable visual acuity and normal macular exams at last FUP

Faure et al. [19]	1	49	M	120	Bilateral macular	(i) Parafoveal retinal graying (ii) Bilateral crystal and pigment deposits	(i) Foveal hyper-AF combined to areas of hypo-AF due to retinal pigment epithelium disruption (RE > LE)	(i) Subfoveal iRORA and nasal juxtafoveal cRORA (RE) (ii) iRORA (LE) (iii) Disruption of inner retina OU	Absent	(i) ff-ERG: dark- and light-adapted responses reduced (ii) Multifocal-ERG: impaired responses to the central hexagons	(i) Nearly 24 m (ii) Stable clinical picture after ritonavir cessation (iii) New pattern of pigment deposits

Mesquita et al. [20]	1	53	M	120	Bilateral retinal and macular	Diffuse bilateral RPE atrophy	Patchy confluent areas of hypo-AF surrounded by hyper-AF	(i) cRORA (ii) FTMH (LE) (iii) ERM (LE)	Absent	—	—

Louie and Jones [21]	1	53	M	84	Bilateral retinal and macular	(i) Bilateral subtle annular pattern of retinal RPE around the fovea (ii) Bilateral yellowish-white chorioretinal lesions and bone spicule-like pigmentary changes in the midperipheral retina	(i) Annular hyper-AF in the parafoveal region consistent with bull's eye maculopathy (ii) Hyper-AF in the areas of the bone spicule-like pigment changes (iii) Smaller patches of hyper-AF along the far inferotemporal arcades	(i) Annular parafoveal iORA (ii) Thickened hyperreflectivity of the subfoveal ellipsoid zone with relative attenuation centrally (iii) Punctate hyperreflective flecks within the ellipsoid zone eccentrically (precursor to crystalline deposits)	Present	ff-ERG: reduced rod- and cone-mediated responses in amplitude and timing, with cones more impaired than rods	(i) More than 24 m (ii) Stable visual acuity, imaging, and functional tests (with ritonavir discontinuation)

cORA = complete outer retinal atrophy; cRORA = complete retinal pigment epithelium and outer retinal atrophy; ERG = electroretinography; ERM = epiretinal membrane; FAF = fundus autofluorescence; ff-ERG = full field electroretinography; FUP = follow-up; FTMH = full-thickness macular hole; hyper-AF = hyper-autofluorescence; hypo-AF = hypo-autofluorescence; iRORA = incomplete outer retinal atrophy; LE = left eye; m = months; M = male; OCT = optical coherence tomography; OU = oculus uterque; PERG = pattern electroretinography; RE = right eye; RPE = retinal pigment epithelium; y = years.

## Data Availability

The data and findings supporting this systematic review are from previous case reports, which have been cited. The processed data are available from the corresponding author upon request.

## Appendix

The case report titled “Bull's eye maculopathy in an HIV-positive patient receiving ritonavir” by Non et al. [37] was excluded for the lack of sufficient clinical details and imaging.

The authors report a case of bull's eye maculopathy pattern in an HIV-positive patient, being treated with ritonavir for 13 years. They describe temporal optic nerve head atrophy in the left eye and areas of macular retinal pigment epithelium (RPE) atrophy with RPE mottling in both eyes. However, they do not provide any fundus autofluorescence image, to confirm the presence of macular atrophy. On the OCT scan, the choroidal hypertransmission is not assessable and the image quality is not high enough to determine the RPE and outer retina status. The diagnosis a previous arterial vascular accident of the retina (temporal nerve head pallor along with central retinal thinning) in the left eye cannot be clearly ruled out on the basis of the information supplied by the authors.
